# Mountaintops phylogeography: A case study using small mammals from the Andes and the coast of central Chile

**DOI:** 10.1371/journal.pone.0180231

**Published:** 2017-07-03

**Authors:** R. Eduardo Palma, Pablo Gutiérrez-Tapia, Juan F. González, Dusan Boric-Bargetto, Fernando Torres-Pérez

**Affiliations:** 1 Departamento de Ecología, Pontificia Universidad Católica de Chile, Santiago, Chile; 2 Laboratorio de Ecología y Biodiversidad, Facultad de Química y Biología, Universidad de Santiago de Chile, Santiago, Chile; 3 Instituto de Biología, Pontificia Universidad Católica de Valparaíso, Valparaíso, Chile; National Cheng Kung University, TAIWAN

## Abstract

We evaluated if two sigmodontine rodent taxa (*Abrothrix olivacea* and *Phyllotis darwini*) from the Andes and Coastal mountaintops of central Chile, experienced distributional shifts due to altitudinal movements of habitat and climate change during and after the Last Glacial Maximum (LGM). We tested the hypothesis that during LGM populations of both species experienced altitudinal shifts from the Andes to the lowlands and the coastal Cordillera, and then range retractions during interglacial towards higher elevations in the Andes. These distributional shifts may have left remnants populations on the mountaintops. We evaluated the occurrence of intraspecific lineages for each species, to construct distribution models at LGM and at present, as extreme climatic conditions for each lineage. Differences in distribution between extreme climatic conditions were interpreted as post-glacial distributional shifts. *Abrothrix olivacea* displayed a lineage with shared sequences between both mountain systems, whereas a second lineage was restricted to the Andes. A similar scenario of panmictic unit in the past was recovered for *A*. *olivacea* in the Andes, along with an additional unit that included localities from the rest of its distribution. For *P*. *darwini*, both lineages recovered were distributed in coastal and Andean mountain ranges at present as well, and structuring analyses for this species recovered coastal and Andean localities as panmictic units in the past. Niche modeling depicted differential postglacial expansions in the recovered lineages. Results suggest that historical events such as LGM triggered the descending of populations to Andean refuge areas (one of the *A*. *olivacea*’s lineages), to the lowlands, and to the coastal Cordillera. Backward movements of populations after glacial retreats may have left isolates on mountaintops of the coastal Cordillera, suggesting that current species distribution would be the outcome of climate change and habitat reconfiguration after LGM.

## Introduction

The Pleistocene, particularly the last .9 Mya, characterized by worldwide climatic changes associated with glacial cycles that increased in amplitude, forcing species to shift their ranges, and subsequently impacting the structure of their populations. There have been studies proposing that under glacial-dominated scenarios, species at higher latitudes experienced strong demographic and genetic changes in their populations [1]. In boreal communities, organisms contracted their distributions to refugia during glacial maxima, and then expanded into newly available habitats after glacial retreat (e.g. [2,3]. In South America, Pleistocene glacial events would have had severe effects on populations associated with Andean mountains, where ice sheets and permafrost were focused on the southern cone of the continent [4–7]. Populations inhabiting higher latitudes would have suffered local extinctions, expansions and retractions following Quaternary glacial oscillations [2,8,9].

Montane regions are of particular interest when assessing species’ responses to historical climate oscillations, because they can cause favorable environments for a species to shift, contract, or expand its geographic range along not only elevational [1,2] but also latitudinal gradients [10]. During climatic fluctuations, mountain populations may experience alternating periods of isolation and connectivity, with for example, range expansion during glacial periods and range contractions during warmer interglacials [11–16].

Montane environments are a major component of the Chilean biogeography, particularly in central Chile where a Mediterranean ecosystem is located along the western margin of the Andes between 30–37° S [17]. Species diversity for the latter area has been hypothesized by different speciation modes, at least for lizards and rodents, as a result of differential interaction among mountain geography, Quaternary glaciations and ecological features: lizards differentiation would occur in the valley during glaciations and also in the mountains during interglacial, whereas rodents would differentiate only in the valley during glaciations. [18]. In addition, this ecosystem is characterized by highly heterogeneous vegetation mosaic and major vegetation types including dry, xerophytic thorn scrub dominated by summer deciduous shrubs and succulents. The mesic communities of this ecosystem are dominated by evergreen sclerophyllous trees in the coastal and Andean foothills, and the forests are dominated by winter-deciduous trees in the southern edge of the region. The southern border of the Mediterranean ecoregion is the Bio-Bío River (37° S), whereas the northern limit is the Atacama Desert in the Copiapó region (27° S).

Biogeographers, when intending to explain disjunct patterns of species distribution on the Cordillera de los Andes and the Cordillera de la Costa (that run in parallel along the country), hypothesize that the disjunction would have occurred by upward shifts and allopatric divergence during interglacial periods, followed by downward shifts and admixture during glacial phases [19]of the Mediterranean ecoregion. Geological and glaciological data on the Last Glacial Maximum (LGM) during Pleistocene times, demonstrated that about two thirds of the Temperate and Patagonian forests were reached by glaciers [4,20,21]. Towards the north, ice masses advanced throughout the Cordillera de los Andes, and in central Chile descended to around 1,100–1,300 m [7,22,23]. These ice masses triggered a local drop of temperatures of about 6–7° C and an increase in the rainfall [24–26]. As a consequence, the Andean vegetational belts shift downwards, to the central valley depression [27–31]. Following glacial cycles, a warmer climate prevailed with a subsequent shift of the vegetational belts upwards not only to the Andes, but also to Coastal altitudes. These events created true “biogeographic islands” at different localities on the top of the coastal mountain in central Chile, now hosting disjunct biota whose main ranges are at similar altitudes in the Andes.

To date, there are few studies about habitat shifts of biota between both mountain systems in Mediterranean Chile from a genetic perspective [32,33]. Most studies have focused on vegetation showing the floristic affinities between the high-andean biotas of the coastal and Andes mountains [34,35], and probable terrestrial corridors from the Andes to coastal mountain due to the descent of temperatures and vegetational habitats during last glaciation [35]. However, there are some studies using molecular tools for some of the coniferous species of the southern flora of Chile (37–43° S), particularly for the coastal mountaintops that have a distribution that is mainly Andean (i.e. *Araucaria araucana* [pehuén]; *Fitzroya cupressoides* [the “alerce”]; *Austrocedrus chilensis* [“ciprés de la cordillera”] [19]. In all the latter case studies a marked fragmentation of coastal populations has been reported with strong genetic segregation of populations between the Coast and Andean taxa [36–38].

The major goal of this paper was to investigate the distribution range dynamics of small mammals across the Andes and coastal mountaintops of central Chile, and if those glacial range shifts are consistent with the displacements of their habitat towards the lowlands and the coastal mountains. To that goal, we used two sigmodontine rodent taxa as study models, each inhabiting both mountain ranges: *Phyllotis darwini* and *Abrothrix olivacea*. *Phyllotis darwini* is an endemic species of Mediterranean Chile and altitudinally it is found between the coast up to 2,000 m [39]. *Abrothrix olivacea*, on the other hand, has a wide distributional range, from southern Peru downward to the Patagonia of Chile and Argentina, and altitudinally it is also found up to 2,000 m [40,41]. Therefore, the major hypothesis to evaluate in this paper is that during LGM populations of *P*. *darwini* and *A*. *olivacea* experienced altitudinal shifts due to displacement of their habitats from the Andes to the coast through the lowlands. Current distribution of intraspecific lineages must have been largely determined by postglacial climate changes and habitat reconfiguration. We predict that contraction events during the interglacial may have left remnants of these two rodent species populations on the coastal Cordillera of central Chile, leaving disjunct populations on the mountaintops of the Cordillera de la Costa and Cordillera de los Andes. The expected signatures of this process are i) lineages currently distributed in the Andes were distributed at lower altitudes on this mountain range during LGM conditions, and probably expanded through lowlands and coastal Cordillera, ii) given that intraspecific lineages in both species are older than LGM [42], we expect to recover shared haplotypes between Andes and coastal Cordillera, as a consequence of rodent’s habitat displacement during Pleistocene, and iii) we expect to find higher genetic diversity in refugial or source areas, and a lower diversity in recently expanded populations [1,2]. We should discard our hypothesis if those signatures are not demonstrated, i.e. lineages currently distributed at the Andes were not distributed at lower altitudes, genetic diversity in postglacial expansion areas is not lower than in source areas and/or both species show private haplotypes in each mountain range. *Phyllotis darwini* is a species restricted to Mediterranean Chile in semi-xeric, open, and middle elevation areas, whereas *A*. *olivacea* is confined to bushy matorral zones widely distributed from southern Perú throughout Chile southward to Patagonia. For the above reasons, we additionally predict that *P*. *darwini* will have a less phylogeographic structured pattern through both mountain systems in central (Mediterranean) Chile, than *A*. *olivacea* that is more confined to bushy-matorral areas of central Chile, and characterized for having a structured phylogeographic pattern along its geographic range, where several subspecies have been recognized [41]. Therefore, to address the major goal of this paper we analyzed the phylogenetic and population structure, the climatic niche and distributional shifts since LGM on the Andean and coastal populations for the two sigmodontine species *P*. *darwini* and *A*. *olivacea*. We used mitochondrial and nuclear markers analyzed from a phylogeographic and niche distribution model approaches.

## Materials and methods

### Study area and taxon sampling

**Fig 1:**
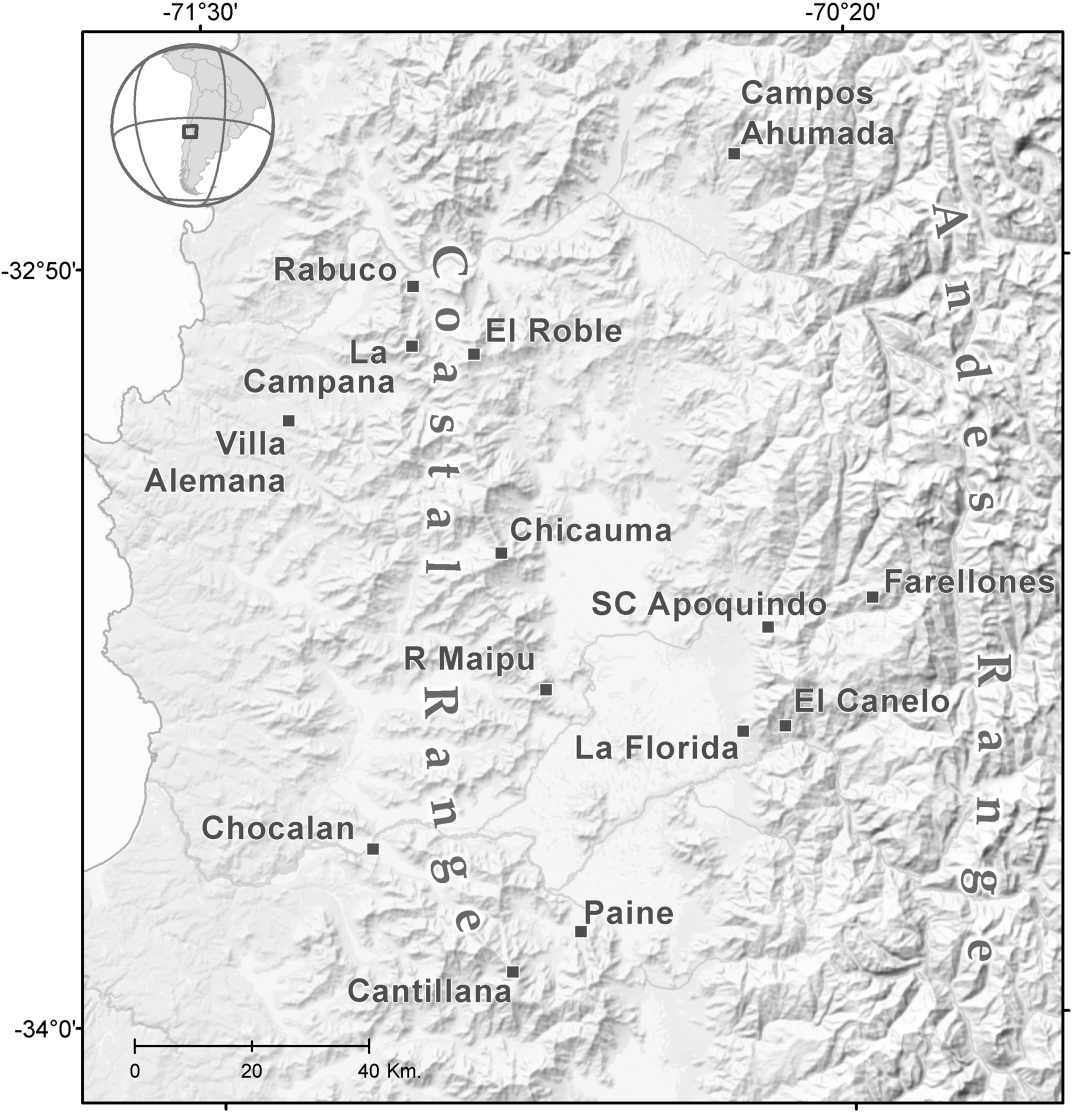
Sampling localities in central Chile. Map showing the localities sampled in central Chile, from the coast, central valley and Andean areas.

We used vertebrate animals (the sigmodontine rodents *Abrothrix olivacea*, *Phyllotis darwini*) which were sacrificed via overdoses of isofluorane and cervical dislocation. These procedures were authorized by the Animal Care and Use Committee of the School of Biological Sciences at Pontificia Universidad Católica de Chile. Field permits were granted by Corporación Nacional Forestal (CONAF, Chile) and Servicio Agrícola y Ganadero (SAG, Chile)." We sampled a complex of localities between the Valparaíso and Metropolitana of Santiago regions encompassing coastal, valley, pre Andean and Andean areas between 32 and 33° S in Mediterranean Chile (see Fig 1 for the study area, and S1 Table for a detailed list of localities). Fourteen localities were sampled in central Chile, four of which were coastal (Cerro La Campana, Cerro El Roble, Altos de Chicauma, Altos de Cantillana), six were from central valley (Villa Alemana, Rinconada de Maipú, Melipilla, Rabuco, La Florida and Paine), and 4 were Andean localities (Farellones, Campos Ahumada, San Carlos de Apoquindo and El Canelo). We trapped a total of 141 mice of which 79 were *A*. *olivacea* and 62 were *P*. *darwini*. Rodent trapping was performed with Sherman traps (8 x 9 x 23 cm) using a mixture of oat and canned fish as bait. Specimens were sacrificed in the field via cervical dislocation previously anesthetized with isofluorane. For each specimen the heart, kidney, spleen, liver and lung were extracted and stored either in ethanol or liquid nitrogen. We followed established safety guidelines for small mammal captures and processing according to the Center for Disease Control and Prevention (CDC) protocols [43], American Society of Mammalogists (ASM) safety guidelines for mammalogists from Hantavirus [44], ASM guidelines for the use of wild mammals in research [45], and the Bioethical Protocols established from the Facultad de Ciencias Biológicas, Pontificia Universidad Católica de Chile. Voucher specimens were deposited in the Colección de Flora y Fauna “Profesor Patricio Sánchez Reyes” (SSUC), Departamento de Ecología, Pontificia Universidad Católica de Chile, Santiago, Chile, and in the Museum of Southwestern Biology (MSB), Department of Biology, University of New Mexico, Albuquerque, New Mexico. Tissues and other data associated with each specimen were cross-referenced directly to each voucher specimen and stored in the collection using a special field catalog number, the NK number used by the SSUC and MSB. A detailed list of the specimens sequenced per locality is given in S1 Table.

### PCR and sequencing protocols

DNA was extracted from frozen liver samples treated with the Wizard Genomic DNA Purification Kit (Promega, Madison, Wisconsin). We amplified via PCR a region of 486 and 574 bp of the mitochondrial DNA (mtDNA) D-LOOP for *A*. *olivacea* and *P*. *darwini*, respectively. We amplified 79 specimens of *A*. *olivacea* and 62 of *P*. *darwini* using primers LBE08 and 12S1 for *A*. *olivacea* [41], and 283F and 282R for *P*. *darwini* [46]. In addition, we amplified a region of 680 bp for *A*. *olivacea* and 584 bp for *P*. *darwini* of the intron 7 of the nuclear b-fibrinogen gene (FGB) for 30 specimens of *A*. *olivacea* and 23 *P*. *darwini* using primers β17-mammL and βfib-mammU [47]; see S1 Table for FGB gene sequenced localities; the specimens sequenced for the FGB gene were chosen taking into account representative individuals from each locality. We followed previously reported thermal protocols to amplify the D-LOOP for *A*. *olivacea* [41], and *P*. *darwini* [46]. Double- stranded polymerase chain reaction products were purified with QIAquik PCR (Qiagen Inc, Valencia, California). BigDye cycle sequencing [48] was performed using primers LBE 08 and 12S1 for *A*. *olivacea*, 283F and 282R for *P*. *darwini*, and the b17- mammL and bfib-mammU for FGB. Cycle sequencing clean up was performed using a QIAquick PCR purification kit (QIAGEN, Inc.) and directly sequenced in ABI DNA 3700 sequencers at Macrogen, Inc. (Geumcheon-Gu, Seoul, Republic of Korea). Sequences were aligned using the CLUSTAL_X program [49] and by eye. All sequences have been deposited in GenBank under accession numbers KT383308-KT383364 (D-LOOP HV2, *Phyllotis darwini*), KT383365-KT383393 (FGB, *P*. *darwini*), KT600174-KT600239 (D-LOOP HV1, *A*. *olivacea*), KT600240-600271 (FGB, *A*. *olivacea*); see S1 Table.

### Phylogenetic analyses

Phylogenetic analyses for both, the D-LOOP and FGB molecular markers as well as for the concatenated matrix were conducted on each haplotype matrix using maximum likelihood (ML) through the online platform PhyML 3.0 [50]. Phylogenetic trees were rooted with the outgroup criterion using the sister species of *P*. *darwini*, *Phyllotis magister* [51] and *A*. *olivacea tarapacensis* for *A*. *olivacea* [41]. The reason why we used the sister species in one case (*P*. *magister*) and a subspecies in the other (*A*. *o*. *tarapacensis*) is that *P*. *darwini* has a more restricted geographic distribution occurring just in the Mediterranean ecoregion, central Chile, and no subspecies are recognized in its range. *Abrothrix olivacea* instead, characterizes for having an extense distribution encompassing a geographic zone that goes from southern Peru to the Patagonia of Chile and Argentina, and several subspecies have been recognized in its distribution [41]. We selected the best-fitting model of nucleotide substitution using the corrected Akaike Information Criterion (AICc)[52] implemented in the program jmodelTest 2 [53]. Support for the nodes was evaluated with 1,000 bootstrap replicates [54]. Sequences were also analyzed in a Bayesian framework to estimate the nodes in a given tree topology. For the D-LOOP matrix 200 millions of iterations were performed, sampling every 1,000 trees to assure that successive samples were independent. The first 20 millions of iterations, meaning the first 20,000 trees of the sample were removed to avoid including trees before convergence of the Markov Chain. For the concatenated matrix (D-LOOP-FGB) 500 millions of iterations were run, sampling every 1,000 trees and burning the first 50 millions of iterations, meaning the first 50,000 trees of the sample were removed to avoid including trees before convergence. Given that we used two independent molecular markers, we applied a general likelihood-based mixture model (MM) [55,56], based on the general time-reversible (GTR) model [57] of sequence evolution. This model accommodates cases in which different sites in the alignment evolved in qualitatively distinct ways but does not require prior knowledge of these patterns or partitioning data. These analyses were conducted using the BAYES PHYLOGENIES software (http://www.evolution.rdg.ac.uk/SoftwareMain.html). To find the best mixture model of evolution we estimated the number of GTR matrices by using a reversible-jump Markov Chain Monte Carlo method RJMCMC [58]. The RJMCMC visits the different mixtures of GTR matrices in proportion to their posterior probabilities, “jumping” from simple to complex models or vice-versa, making a direct estimate of the support of 1GTR, 2GTR, 3GTR, and so on. Only the combination of matrices with the fewest number of parameters that significantly increased the likelihood was used (1GTR + Γ for D-LOOP data; 2GTR + Γ for concatenated data) for *A*. *olivacea* and *P*. *darwini* to compute a 50% majority rule consensus tree. The percentage of samples that recovered any particular clade on this tree represents the posterior probability of that clade; these are the p values, and p ≥ 95% was considered evidence of significant support for a clade [59].

### Population genetic structure analyses

We used the DNASP v 5.10.01 software to describe the genetic diversity in all groups and the complete data set. We calculated the number of haplotypes (Nh), the haplotype diversity (Hd), the nucleotide diversity pi (the average number of pairwise nucleotide differences per site), and the segregating sites (S). We also assessed demographic history of the mountaintop groups by performing Fu’s Fs neutrality test statistics (Fu, 1997). To evaluate population structure for each species, we used GENELAND v. 1.0.7 [60] in the R-Package [61], which implements a population statistical model with Bayesian inference (BI) in a set of georeferenced individuals with DNA sequences data (http://www2.imm.dtu.dk/~gigu/Geneland/#). This model locates genetic discontinuities between populations of geo-referenced genotypes, considering the uncertain localization of the sampled individuals. The number of clusters was determined by running MCMC (Markov chain Monte Carlo) iterations five times, allowing *K* (i.e. the most probable number of populations) to vary, with the following parameters: 25 x 10^6^ MCMC iterations, maximum rate of the Poisson process fixed to 80 for *A*. *olivacea* and 60 for *P*. *darwini* (the minimum K = 1, maximum K = 10, values that allow us to explore a wide potential number of populations, and considering the maximum spatial subdivision in the latitudinal range). The maximum number of nuclei in the Poisson-Voronoi tessellation was fixed to 240 for *A*. *olivacea* and 180 for *P*. *darwini* (3 x maximum rate was previously suggested) [62]. After inferring the number of populations in the data set from these five runs, the MCMC was run 30 times with *K* fixed to the inferred number of clusters, with the other parameters the same as above. The mean logarithm of the posterior probability was calculated for each of the 30 runs and the posterior probability of population membership for each pixel of the spatial domain was then computed for the three runs with the highest values.

To establish the relationships between sequences, we constructed a network using the Neighbor-Net [63] distances transformation and equal angle splits transformation [64]. The Neighbor-Net is a distance methodology that considers the complete sequences using the neighbor net algoritm. Splits computed from the data are represented as parallel edges rather than single branches, allowing visualization of ambiguous and conflicting signals in the data set providing an implicit representation of evolutionary history [65].

### Climatic niche models

#### Distribution models

We modeled the climatic niche of each intraspecific lineage to approximate the whole species’ current distribution, and its distribution during the LGM under the assumptions that: (1) climate is an important factor driving the species distribution; (2) the climatic niche of species remained conserved between the LGM and present time, and (3) overlapped lineage distribution ranges will approach the whole species geographic range during a specific time frame. The latter assumption was tested by overlapping distribution models of each intraspecific lineage, at current conditions, in order to approach the full species distributional range, as the sum of ranges estimated for each lineage. The resultant distributional range was roughly contrasted with another model built for the whole species without considering phylogenetic structure.

The climatic niches were reconstructed using the methodology of ecological niche modeling, where environmental data are extracted from occurrence records and random points (represented by geographic coordinates). Habitat suitability was evaluated across the landscape using specific algorithms [66]. The current models were then projected on the climatic reconstructions of the LGM. For occurrence records, we used our unique sampling localities. In addition to full geographic distribution models for each species, we built climatic models for each major lineage recovered in the intraspecific phylogenies following the same approach. As a test of consistency we overlapped the lineage distribution models for the lineages of each species, to compare it to the full species distribution models.

The current climate was represented by bioclimatic variables from the WorldClim dataset v. 1.4 (http://www.worldclim.org/; [67]) that are derived from monthly temperature and precipitation data, and represent biologically meaningful aspects of local climate [68,69]. For environmental layers representing the climatic conditions of the LGM, we used ocean–atmosphere simulations [70] available through the Paleoclimatic Modeling Intercomparison Project [71]; we used two models that have been previously downscaled for the purpose of ecological niche modeling [68]: Community Climate System Model v. 3 (CCSM; [72]) and the Model for Interdisciplinary Research on Climate v. 3.2 (MIROC; [73]).

Climatic niche models were built in the software package MAXENT v. 3.2.1 [74], a program that calculates relative probabilities of the species presence in the defined geographic space, with high probabilities indicating suitable environmental conditions for the species [75]. Trapping coordinates of each individual captured for DNA extraction were used as presence points. We used the default parameters in MAXENT (500 maximum iterations, convergence threshold of 0.00001, regularization multiplier of 1, and 10,000 background points) with the application of random seed and logistic probabilities for the output [76]. We masked our models to four altitudinal categories resuming both, the abrupt altitudinal clines characteristic of central Chile, and some known altitudinal distribution limits for several vertebrate taxa in this area [18]. This procedure was conducted because reducing the climatic variation being modeled to that which exists within a geographically realistic area improves model accuracy and reduces problems with extrapolation [77–79]. We ran 10 replicates for each model, and an average model was presented using logistic probability classes of climatic niche suitability. The presence—absence map was determined using the ‘maximum training sensitivity plus specificity logistic threshold’ where the omission error of all occurrence records is set to zero (i.e. locations of all occurrence records are predicted as ‘suitable’). Inside this suitability area, we show the 50% highest logistic probability values observed between the maximum training sensitivity plus specificity logistic threshold and the maximum observed logistic value, in order to depict the areas with highest logistic values (red areas). We used the receiver operating characteristic for its area under the curve (AUC) value to evaluate the model performance [80,81]. AUC values ranged from 0.5 for a random prediction to 1 for perfect prediction [75].

## Results

For the D-LOOP region, we sequenced 79 specimens of *A*. *olivacea* of which 27 were from the Andes, 29 from the valley and 23 from the coast, whereas for *P*. *darwini* we sequenced 62 specimens of of which 27 were from the Andes and 35 from the coast. The data on the D-LOOP sequence variability for both species are presented in Table 1 for each mountain system. For *A*. *olivacea* we also give the data for the valley (we did not have samples for the valley in *P*. *darwini*). We observe that for both species there is a higher number of haplotypes in the Andes than in the coast, which is remarkably notorious for *P*. *darwini* (Table 1); at the same time the haplotype diversity (Hd) is higher in the Andes mountains than in the coast (and the valley when is the case) for both species. Regarding the polymorphic sites (S) these are also higher for both species in the Andes than in the coast (and the valley for *A*. *olivacea*). Finally, the data show that the nucletide diversity, pi value, is very low for samples of *A*. *olivacea* of the coast and the valley if compared to samples of the Andes for the same species; this value is also higher for the Andes than in the coast for *P*. *darwini* (Table 1). Fu’s test values for *A*. *olivacea* were significantly different from zero for the valley (-1.475) and the Coast localities (-3.041), indicating population expansion, whereas for the Andes it was not significantly different from zero suggesting a population in equilibrium (-0.654). Fu’s neutrality test statistics for *P*. *darwini* was negative and significantly different from zero for the Andes (- 3.12) indicating that the null hypothesis of population equilibrium is rejected in favor of a population expansion, whereas for the Coast was positive (3.437) and significantly different from zero, suggesting a population bottleneck due to significant deficiency of alleles.

**Table 1:** D-LOOP sequence variability data for the Coast, Valley and the Andes in *Abrothrix olivacea* and the Coast and the Andes for *Phyllotis darwini*. Abreviations mean Nh = number of haplotypes, Hd = haplotype diversity, S = polymorphic sites and pi = nucleotide diversity.

	*Abrothrix olivacea*				*Phyllotis darwini*			
	Nh	Hd	S	pi	Nh	Hd	S	pi
Coast	9	0.727	14	0.004	13	0.780	51	0.023
Valley	7	0.603	10	0.003				
Andes	12	0.895	20	0.013	22	0.983	59	0.037

**Fig 2:**
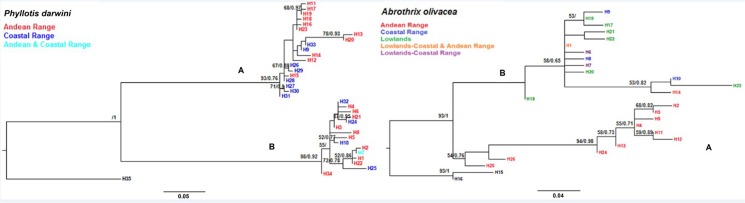
Maximum likelihood (ML) and Bayesian inference (BI) haplotype trees based on D-LOOP sequences of *Phyllotis darwini* and *Abrothrix olivacea*. Haplotype phylogenetic trees representing the intraspecific relationships of *Phyllotis darwini* and *Abrothrix olivacea* from central Chile mountaintop and lowland areas. Numbers on the nodes represent the posterior probability and 1,000 bootstrap support values. Color labels on the trees represent mountaintop and/or lowland haplotypes as specified inside the figure. Outgroup for the phylogenies are recovered at the bottom of each phylogeny represented by *Phyllotis magister* and *Abrothrix olivacea tarapacensis*. For *P*. *darwini* A = lineage A (coastal and Andean), B = lineage B (coastal and Andean); For *A*. *olivacea* = A = lineage A (Andean), B = lineage B (coastal and valley).

**Fig 3:**
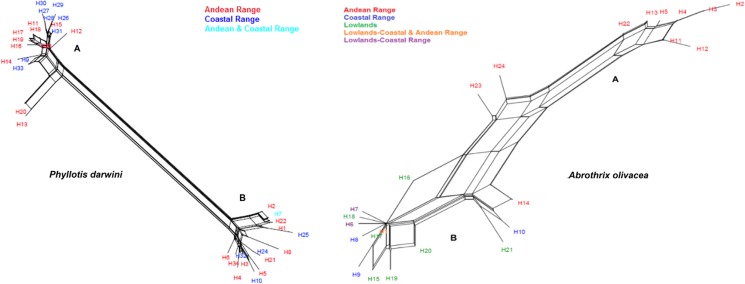
Neighbor-Net of D-LOOP sequence haplotypes of *P*. *darwini* and *A*. *olivacea*. Labels for haplotypes on the network are explained inside the figure.

The D-LOOP based intraspecific phylogeny for *A*. *olivacea* was similar for both, ML and BI, thus we show a single tree (Fig 2). For this species we observed a well-supported split between two major clusters. One of them is constituted by haplotypes sampled exclusively in Andean localities (e.g. Farellones, San Carlos de Apoquindo, Campos Ahumada (lineage A, red haplotypes, Fig 2). The other major group mostly included haplotypes sampled in the coastal range or the lowlands (blue and green haplotypes, Fig 2), mixed with some haplotypes from the Andes (red haplotypes). However, in the Andean phylogroup (lineage A) we did not obtain any coastal sequence for *A*. *olivacea* (Fig 2). As for *P*. *darwini*, we also recovered a well-supported dichotomy of two differentiated phylogroups, although we could not recognize any of the clusters strictly associated to a specific mountain range. In fact, the largest phylogroup (lineage A, Fig 2) reunited coastal (e.g. Cantillana, El Roble, Chicauma; blue haplotypes) and Andean localities (e.g. Farellones, Campos Ahumada; red haplotypes); a similar situation occurred for lineage B. Even though *P*. *darwini’s* lineage A is distributed in both mountain ranges, it is noteworthy that this lineage is distributed exclusively in localities above 1,500 m altitude. This pattern has been previously reported for the species, with a completely different sample set using only D-LOOP mitochondrial sequences [42]. The Neighbor-Net analysis, on the other hand, showed similar patterns of divergence to that recovered through the intraspecific phylogenies for the Coastal cordillera (blue), the lowlands (green) and the Andean (red) haplogroups for *A*. *olivacea*, and between the Coastal (blue) and Andean (red) cordilleras for *P*. *darwini* (Fig 3).

**Fig 4:**
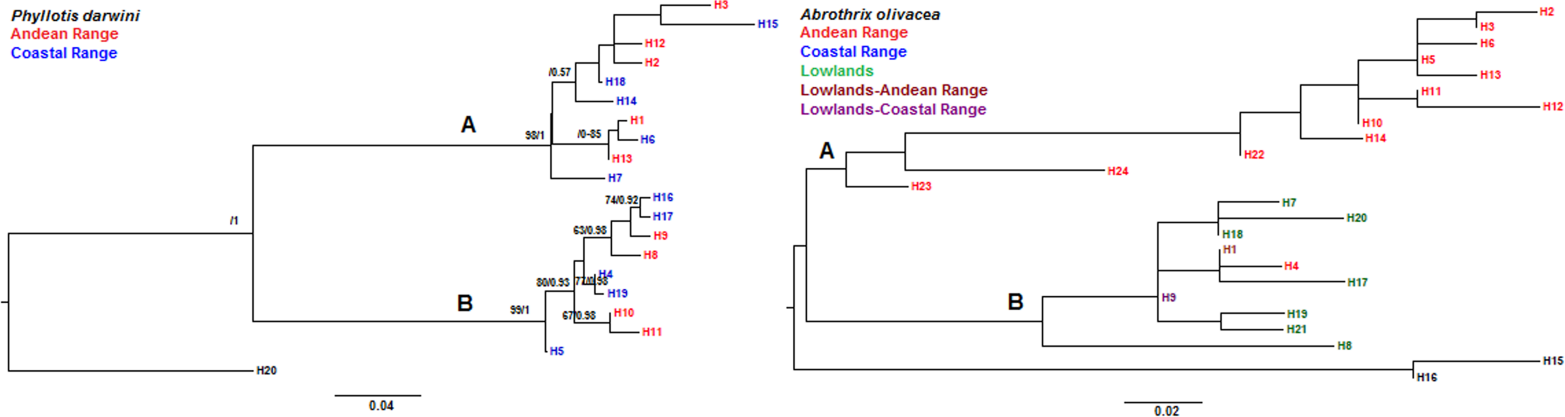
BI and ML haplotype trees based on the combined D-LOOP-FGB sequences for *P*. *darwini* and *A*. *olivacea*. The haplotype trees represent the intraspecific relationships of *P*. *darwini* and *A*. *olivacea* from central Chile mountaintop and lowland areas (see S2 and S3 Tables for the geographic location of haplotypes of *A*. *olivacea* and *P*. *darwini*, respectively). Numbers on the nodes represent the posterior probability and 1,000 bootstrap support values. Color labels on the trees represent mountaintop and/or lowland haplotypes as specified inside the figure. For *P*. *darwini* A = lineage A (coastal and Andean), B = lineage B (coastal and Andean); For *A*. *olivacea* = A = lineage A (Andean), B = lineage B (lowlands-Andean and lowlands).

A similar topology to that of D-LOOP was obtained for *A*. *olivacea* when analyzing phylogenetically the concatenated D-LOOP and FGB haplotype sequences (Fig 4), in which it is clear the dichotomy between a strictly Andean phylogroup (lineage A) and a mixed phylogroup (lineage B). We show the concatenated instead of the FGB tree, since the latter showed a very low variability, where is not easy to recognize some pattern regarding the relationships between the coastal and the Andean haplotypes. Alternatively, the combined D-LOOP/FGB phylogenetic analysis (ML and BI) for *P*. *darwini* showed a similar topology to that obtained with D-LOOP, recognizing two well supported phylogroups that combined haplotypes from the coast and the Andes, with lineage A distributed above 1,500 m altitude (Fig 4). FGB haplotypes showed low variation with 21 polymorphic sites for *P*. *darwini* and only 5 for *A*. *olivacea*. The only big difference is that lineage A of *A*. *olivacea* would not be exclusive of the Andes because that lineage also includes three sequences of the central valley (Fig 4).

**Fig 5:**
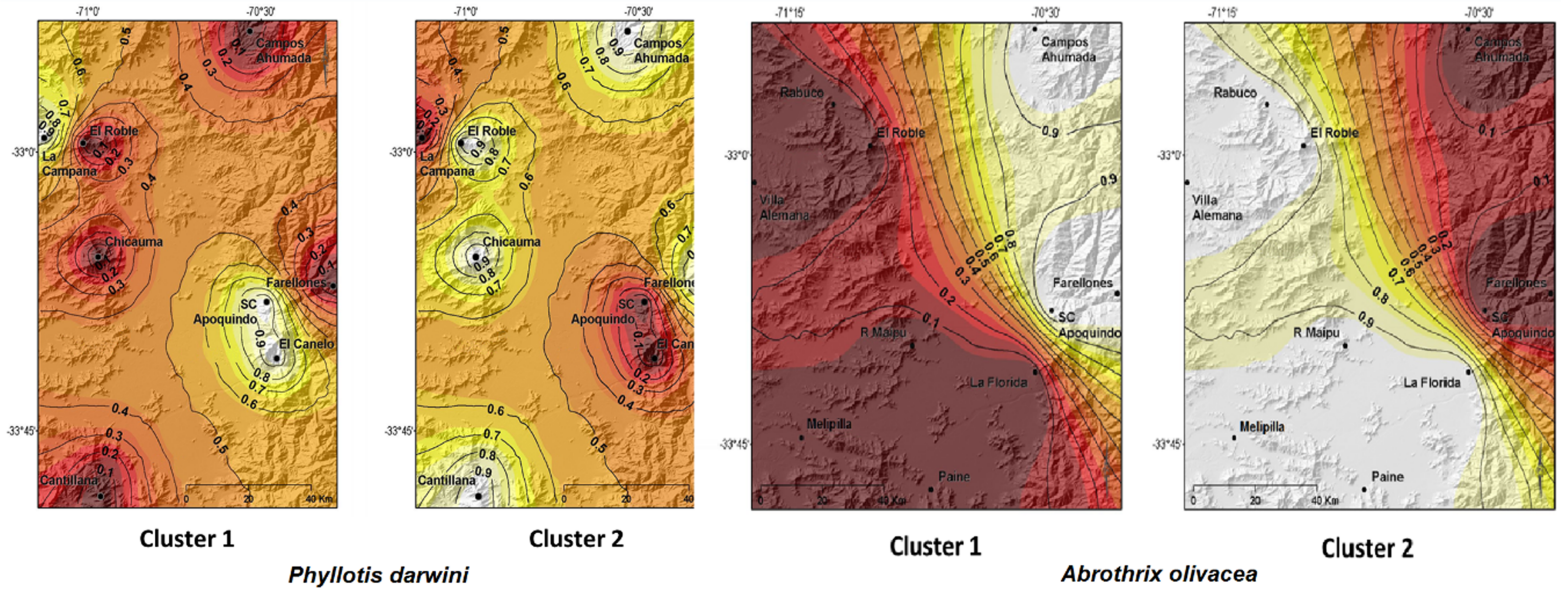
GENELAND analysis with posterior probability isoclines for *P*. *darwini* and *A*. *olivacea*. The genetic structuring analysis denotes the extent of genetic landscapes for the two clusters recovered in *P*. *darwini* and two clusters for *A*. *olivacea*. Coastal and Andean mountaintops are recovered in the figure. To facilitate interpretation, GENELAND output has been cropped, re-scaled and superimposed over the map of central Chile where this study was conducted for *A*. *olivacea*. Black dots represent localities analyzed in this study. Regions with the greatest probability of inclusion are indicated in white, whereas lower probabilities are represented in increasingly darker coloring.

The GENELAND analyses recovered two clusters for each species (Fig 5 *A*. *olivacea* and *P*. *darwini*). Cluster 1 for *A*. *olivacea* suggested that Andean localities of Campos Ahumada, Farellones and San Carlos de Apoquindo constitute a single population with high probability values as it is shown through the posterior probability isoclines (Fig 5). Cluster 2 for *A*. *olivacea* suggested that lowland coastal areas such as Rabuco and Villa Alemana and El Roble from the Coastal Cordillera belong to a genetic unit together with La Florida, a locality adjacent to the Andes. For *P*. *darwini*, on the other hand, cluster 1 showed that Andean populations of El Canelo and San Carlos de Apoquindo constituted a single genetic unit along with the population of La Campana in the Coastal Cordillera, despite being currently distributed in disjunction. Whereas, for cluster 2 in the same species, coastal mountaintop populations of Chicauma, Cantillana and El Roble seem to have formed a single genetic unit with Andean populations of Campos Ahumada and Farellones (Fig 5).

### Distribution models

**Fig 6:**
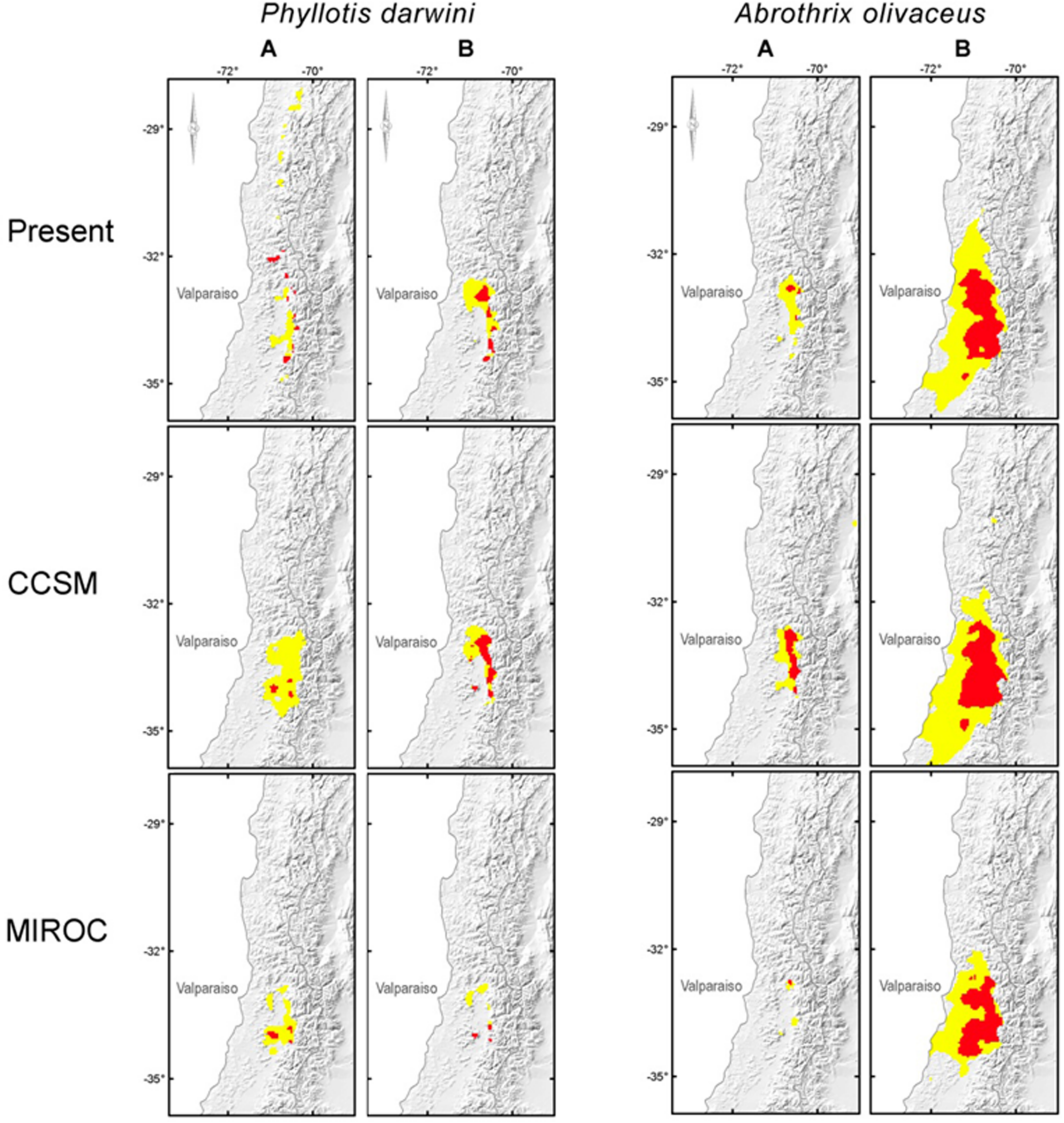
Lineage distribution models for *A*. *olivacea* and *P*. *darwini*. The figure shows the species distribution models for the present, and the LGM climatic scenarios (rows). Models were built independently for *P*. *darwini* and *A*. *olivacea* intraspecific lineages (columns). The yellow color represents suitability areas for the lineages, according to the maximum training sensitivity plus specificity logistic threshold; the red areas represent 50% highest logistic probability value observed between the maximum training sensitivity plus specificity logistic threshold, and the maximum logistic probability value for each model.

In order to test the assumption that overlapped lineage distribution models may approximate whole species distribution models, we compared our estimations of each species distribution range at present from i) all trapping localities, considering whole species as single distributional unit (data not shown), and ii) submodels for trapping localities assigned to different intraspecific lineages as independent distributional units (Fig 6, Tables 2 and 3). The results showed that whole species’ range models are good approximations of the observed distribution range for each species, and also with high model performance (AUC, Table 2). Overlapped lineage distribution models in *P*. *darwini* performed as well as did the whole species model; in the case of *A*. *olivacea*, overlapped lineage models performed even better than the whole species range model. Consequently, both model approaches (whole species and overlapped lineage’s ranges) are good and consistent approximations of current species´ geographic ranges (considering the whole species’ range as the portion of the distribution of *A*. *olivacea* and *P*. *darwini* assessed in this work).

**Table 2:** Area under the curve (AUC) average values for each distribution model. These values represent the Area under the “receiving operating characteristic” curve. AUC value measures the performance of a distribution model, which ranges from 0.5 for a random prediction to 1 for perfect prediction.

MAXENT Model	AUC Average	AUC stdv.
*Phyllotis darwini* whole species range	0.971	0.022
*Phyllotis darwini* A	0.970	0.050
*Phyllotis darwini B*	0.969	0.023
*Abrothrix olivacea* whole specie´s range	0.875	0.046
*Abrothrix olivacea* A	0.916	0.028
*Abrothrix olivacea* B	0.916	0.086

**Table 3:** General description of hypothesized distribution for each lineage with latitudinal extension and orographic characteristics of the suitable areas. Columns are intraspecific lineages and rows represent climatic models for present and LGM conditions (CCSM and MIROC). The last row indicates if a lineage has a stable distribution since LGM until present day, or if its distribution range has changed.

	*P*. *darwini* A	*P*. *darwini* B	*A*. *olivacea* A	*A*. *olivacea* B
**Present**	28° S-35° S	32° S-34° S	32° S to 34° S	31° S-35° S
	Mainly at the Andes and some suitable areas at the coastal cordillera between 32° S-34° S.	Suitable areas at the Andes, and discontinuous distribution at valley and coastal mountain range.	Suitable areas mainly at the Andes; some suitable spots at the coastal mountain range.	Suitable areas at the Andes, valley and coastal mountain range.
**CCSM**	32° S-34° S	32° S-34° S	32° S—34° S	31° S-35° S
	Suitable areas at the valley, Andes and coastal mountain ranges.	Suitable areas at the Andes and discontinuous distribution at the valley and the coastal mountain range.	Suitable areas at the Andes, valley and coastal mountain range.	Suitable areas at the Andes, valley and coastal mountain range.
** **		** **		** **
**MIROC**	32° S-34° S	32° S-34° S	32° S—34° S	32° S-34° S
	Suitable areas at the valley, Andes and coastal mountain ranges.	Suitable areas at the Andes and discontinuous distribution at the valley and the coastal mountain range.	Isolated suitable spots, mainly at low altitude localities in the Andes.	Suitable areas at the Andes, valley and coastal mountain range.

### Range dynamics

#### Phyllotis darwini

Distribution model at current climatic conditions for this species showed that lineage B has suitable areas across the Andes between 32° S and 34° S, and in some spots of the coastal mountain range within the same latitude; the valley is also suitable for this lineage between 32° S and 33° S. The distribution model for *P*. *darwini*’s lineage A is surprisingly well defined across the Andes between 27° S and 35° S; this lineage also displayed suitable spots across the coastal mountain range between 31° S and 34° S, with high logistic probability values (Fig 6, red areas). Lineage A had also suitable areas in the points where both, Andean and coastal mountain ranges are very close to each other and the valley becomes narrow.

Both distribution models for lineage B at LGM (CCSM and MIROC, Fig 6) showed that latitudinal distribution was approximately the same that at present, but high altitude spots at the Andes were absent at those climatic conditions; there is some disagreement between both models: according to the CCSM model, lineage B distribution at the valley and the coastal mountain range was approximately the same that at current conditions. On the other hand, MIROC based distribution model displayed a relictual distribution for lineage B during LGM (Fig 6), restricted to a narrow low altitude fringe at the Andean border, and some isolated populations at coastal mountain range at 32° S and 34° S. On the other hand, differences between current and past distribution models for lineage A in *P*. *darwini* are far more dramatic: CCSM and MIROC based models showed that the broad Andean distribution observed at present was almost absent during LGM, when this lineage had notoriously expanded its distribution towards the valley and coastal mountain range (northern distribution limit for the species could have been located at 32° S at LGM, five latitude degrees south from current northern limit).

#### Abrothrix olivacea

Distribution model at current climatic conditions for this species showed that lineage B had a broad suitable distribution area between 31° S to 35° S, at the Andes, the valley and the coastal mountain range. Meanwhile, lineage A displayed suitable areas at the Andes between 32° S-34° S, and some suitable spots in the coastal mountain range (although, all individuals assigned to this lineage have been sampled at Andean localities).

Hypothesized distribution at LGM for lineage B is almost identical to its current distribution according to both, CCSM and MIROC based distribution models (Fig 6). A very similar behavior was observed for the “Andean” lineage A; the only disagreement occurred in MIROC based distribution models, which showed a smallest suitable area for this lineage at LGM, but with very similar latitudinal and altitudinal distribution. According to this model, it is possible that *A*. *olivacea*’s lineage A may have been restricted to a narrow low altitude fringe at the Andean border. Consequently, the distribution of both lineages in *A*. *olivacea* had remained relatively constant since LGM to present, with probably small postglacial expansions towards the Andes; it is possible that lineage A have had remained distributed exclusively in Andean localities. On the other hand, *P*. *darwini*’s lineage A has notouriously expanded its distribution northwards through the Andean mountain range, and to suitable areas in the valley. Meanwhile, in the coastal mountain range, *P*. *darwini* had contracted its distribution since LGM until present day, leaving just some isolated populations at the top of the Coastal Cordillera.

## Discussion

Our results exhibited an evident split of haplogroups for both species of sigmodontines of the Andes and the Coastal Cordillera in the study area. For *A*. *olivacea* we observed that one of the phylogroups is restricted to Andean localities (lineage A), while the other is distributed in both mountain ranges, some in the valley and some in coastal localities (lineage B). For *P*. *darwini* we also recovered two phylogroups distributed in the Andes and in the Coastal mountain ranges. Although both phylogroups are latitudinally overlapped within the latter species, one of them (lineage A) is restricted to localities above 1,500 m across both mountain ranges but with a broad latitudinal extension across the Andes, whereas the other (lineage B) ranges in the valley and both mountain ranges [42].

We suggest that the biogeographic mechanism that may have triggered the dynamic range shift in the recent evolutionary history of *P*. *darwini* and *A*. *olivacea* was the downwards displacement of the Andean vegetational belts towards the valley, as a consequence of the 7° C drop of temperature driven by the ice advance throughout the Andes of central Chile at the LGM [7,22]. The observed pattern meets our predictions of i) Andean lineages distributed at lower altitudes during LGM compared to its current distribution, and ii) the occurrence of mixed haplotypes between both mountain ranges. Glaciations may have allowed Andean populations to refuge at low altitudes in the Andes mountains and/or in areas free of ice in the Coastal Cordillera. The latter biogeographic scenario would be sustained by the sequence genetic diversity results obtained for both species, (e.g., H, Hd, S) observing a higher genetic diversity for the Andes populations compared to coastal mountaintops, or even for the valley in the situation of *A*. *olivacea*. In the latter scenario, we have the case of populations distributed at lower altitudes, which it would suggest an eventual refuge at lower elevations in the Andes. Lower diversity indexes where then recorded for coastal and valley populations suggesting recent expansions. Fu’s demographic results also show that populations have been in demographic expansion particularly in the case of *P*. *darwini* both for the coast and the Andes, whereas for *A*. *olivacea* these Fu’s values were significant for coastal and valley populations but not for the Andes, where populations remained at lower elevations or refuge areas. GENELAND analyses, suggest different patterns of population displacement for each of the studied species. The latter analyses for *A*. *olivacea* (Fig 5 cluster 1) suggest that high elevation populations of Farellones with low elevation Andean populations of San Carlos de Apoquindo and Campos Ahumada may have constituted panmictic units in the past, with a probably restricted gene flow at present (Fig 2; Fig 5 cluster 1). Thus, altitudinal populations of *A*. *olivacea* might have refuged at lower elevations seeking more optimal conditions due to the temperature drop at the LGM, populations that today occur at different elevations in the Andes. Whereas, for the other studied species, *P*. *darwini*, GENELAND results suggest that populations from the Andes and from the mountains of the Coast conformed a panmictic unit due to the movement between both cordilleras as depicted in clusters 1 and 2 (Fig 5). Thus, our results suggest that mountain populations of *P*. *darwini* and *A*. *olivacea* moved between both cordilleras or altitudinally in the Andes, and that these movements might have been triggered by the biogeographic events that affected the latter mountains during the Pleistocene glacial cycles. However, what evidence do we have of these movements, and when these might have occurred since during Pleistocene several glaciation cycles have been reported?

Niche modeling analyses for both species suggest that there have been lineages with persistent distribution at the valley and the coastal mountain ranges which have slightly expanded their distribution towards the Andes after glacial retreat (*P*. *darwini*’s lineage B and *A*. *olivacea*’s lineage B), whereas, other lineages have dramatically expanded their range exclusively across mountain ranges (*P*. *darwini*’s lineage A). The only exception to our prediction are the private haplotypes in the *A*. *olivacea’* Andean phylogroup, which have apparently remained distributed at low elevation refuges in the Andes, without reaching the coastal mountain. This particular phylogroup would not contribute to the eventual occurrence of shared haplotypes between mountain chains, although this is not in conflict with the downward distributional shift from the Andes to the lowlands during LGM. The fact that *A*. *olivacea* displayed a strictly Andean phylogroup, and another lineage with broad distribution in both mountain systems, suggests that the mixed phylogroup is the result of range displacements between mountain ranges, while the Andean phylogroup appears to have remained restricted to the Andes, even with relictual distribution during glacial cycles (Fig 5, and as is suggested by its hypothesized distribution during LGM according to the MIROC based distribution model). On the other hand, both lineages of *P*. *darwini* are distributed in both mountain ranges (one of them strictly restricted to elevations above 1,500 m with disjunct distribution along both cordilleras). In addition, the lineages in *P*. *darwini* displayed the largest distributional shifts from LGM until current conditions, with a high-altitude lineage which has dramatically expanded its distribution across the mountains. Therefore, we hypothesize that *P*. *darwini*’s current distribution has been determined by at least the last glacial cycle (differential postglacial colonization for each intraspecific lineage), and the origin of their lineages is probably related to ancient range shifts across the mountains.

Why do we have different biogeographic patterns on the mountaintops for the two species of sigmodontine rodents that coexist in central Chile? Our results showed that *P*. *darwini* shares more sequences between both mountain systems compared with *A*. *olivacea*, which possess one lineage that remained in the Andes, even during LGM. *Phyllotis darwini* characterizes for being one of the most ubiquitous species in the semiarid and arid regions of northern and central Chile and it appears to be able of seasonally adjust its resistance to desiccation utilizing seeds and succulents [82,83]. In contrast, *A*. *olivacea* characterizes for preferring habitats with less shrub and greater herbaceous cover [84]. Besides *A*. *olivacea* characterizes by its wide distributional range along Chile, its phylogeography exhibits a clearly structured pattern, suggesting local adaptation of populations at the different environments where the species occurs from semiarid, to Mediterranean and forest environments along the territory [41].

Thus, our hypothesis to explain current patterns on mountaintop populations for both species of sigmodontine mice in central Chile, would rest on historical events as the LGM (and probably former glacial/interglacial transitions) that would have triggered the descent of populations from the Andes to lower elevations and refuge areas in Coastal Cordillera as suggested by our phylogeographic analyses. Further movements of populations backwards after glacial retreats may have followed, leaving, in some cases, population isolates on the mountaintops of the Coastal Cordillera. These “mountain island isolates” occurring mainly at the valley and Coastal Cordillera during LGM, may have recolonized the Andean mountains after glacial retreat, explaining the current pattern of high frequency of shared haplotypes probable between mountain ranges, as well as disjunct distribution across both mountains. The occurrence of a unique strictly Andean lineage (*A*. *olivacea*, A) that has also remained associated to a single mountain range through the last glacial/interglacial transition, strongly agreed with this hypothesis. The only phylogroup which does not satisfy our haplotype admixture prediction is also the one which was unable to reach the Coastal Cordillera during LGM according to our distribution model.

It is possible to argue that the currently observed pattern of haplotype admixture between the Andes and the Coastal Cordillera could be attributed to a more ancient fragmentation process (and not to rodent habitat displacement during Pleistocene), but we sustain that the high consistency between our predictions about geographic distribution of haplotypes and distributional shifts, make our general hypothesis the most parsimonious model to explain the observed lineage distribution pattern. Those results rise the need to investigate this hypothesis in additional vertebrate taxa inhabiting the mountains of central Chile, to evaluate if the well documented vegetational displacements during Pleistocene are in fact a more general biogeographic pattern in the latter area.

## Supporting information

S1 Table Sampling sites and GenBank access information. Specimens analyzed, sampling locations, geographic coordinates, GenBank accesses for both genes, and altitude for each of the locations analyzed. All localities analyzed are in central Chile, please see the study area map (Fig 1). The NK number is a special field catalog number for tissues used by the Colección de Flora y Fauna Patricio Sanchez Reyes, Departamento de Ecología, Pontificia Universidad Católica de Chile, Santiago, Chile, and by the Museum of Southwestern Biology, University of New Mexico, USA; UCK is the new tissue number collection used by the Colección de Flora y Fauna Patricio Sanchez Reyes, Departamento de Ecología, Pontificia Universidad Católica de Chile; EP is the field catalogue of Dr. R. Eduardo Palma, and ER the field catalogue of Dr. Enrique Rodríguez-Serrano.
(DOC)

S2 Table Haplotypes of *Abrothrix olivacea* based on D-LOOP mtDNA sequences. Haplotypes recovered for *Abrothrix olivacea* by sequencing the D-LOOP and the FGB genes (for the latter, we show the haplotypes of the concatenated D-LOOP and FGB matrix). We show the haplotype number, the frequency of that haplotype and the voucher with the abbreviation of the geographic locality (see S1 Table for complete details of each locality). The NK number is a special field catalog number for tissues used by the Colección de Flora y Fauna Patricio Sanchez Reyes, Departamento de Ecología, Pontificia Universidad Católica de Chile, Santiago, Chile, and by the Museum of Southwestern Biology, University of New Mexico, USA; UCK is the new tissue number collection used by the Colección de Flora y Fauna Patricio Sanchez Reyes, Departamento de Ecología, Pontificia Universidad Católica de Chile; EP is the field catalogue of Dr. R. Eduardo Palma, and ER the field catalogue of Dr. Enrique Rodríguez-Serrano.
(DOCX)

S3 Table Haplotypes of *Phyllotis darwini* based on D-LOOP mtDNA sequences. Haplotypes recovered for *Phyllotis darwini* by sequencing the D-LOOP and the FGB genes (for the latter, we show the haplotypes of the concatenated D-LOOP and FGB matrix). We show the haplotype number, the frequency of that haplotype and the voucher with the abbreviation of the geographic locality (see S1 Table for complete details of each locality). The NK number is a special field catalog number for tissues used by the Colección de Flora y Fauna Patricio Sanchez Reyes, Departamento de Ecología, Pontificia Universidad Católica de Chile, Santiago, Chile, and by the Museum of Southwestern Biology, University of New Mexico, USA; UCK is the new tissue number collection used by the Colección de Flora y Fauna Patricio Sanchez Reyes, Departamento de Ecología, Pontificia Universidad Católica de Chile; EP is the field catalogue of Dr. R. Eduardo Palma.
(DOCX)
