# Correction: Mountaintops phylogeography: A case study using small mammals from the Andes and the coast of central Chile

**DOI:** 10.1371/journal.pone.0186031

**Published:** 2017-10-02

**Authors:** The PLOS ONE Staff

There are errors in the Funding section. The publisher apologizes for these errors. The correct funding information is as follows: This work was supported by grants from Fondo Nacional de Ciencia y Tecnología (FONDECYT, Chile) 1100558, 1130467 and 1170761 to REP and 1140929 to FTP, URL: http://www.conicyt.cl/fondecyt/; Grant USA 1555.48 to PGT, and Postdoctoral Project 2015–2016 VRIEA PUCV to DBB. The funders had no role in study design, data collection and analysis, decision to publish, or preparation of the manuscript.
