# Supplementary material for: Mountaintops phylogeography: A case study using small mammals from the Andes and the coast of central Chile
Source: PLoS One. 2017 Jul 3;12(7):e0180231. doi: 10.1371/journal.pone.0180231 (PMC5495339; doi:10.1371/journal.pone.0180231)
Supplement: S1 Table — Specimens analyzed, sampling locations, geographic coordinates, GenBank accesses for both genes, and altitude for each of the locations analyzed. All localities analyzed are in central Chile, please see the study area map (Fig 1). The NK number is a special field catalog number for tissues used by the Colección de Flora y Fauna Patricio Sanchez Reyes, Departamento de Ecología, Pontificia Universidad Católica de Chile, Santiago, Chile, and by the Museum of Southwestern Biology, University of New Mexico, USA; UCK is the new tissue number collection used by the Colección de Flora y Fauna Patricio Sanchez Reyes, Departamento de Ecología, Pontificia Universidad Católica de Chile; EP is the field catalogue of Dr. R. Eduardo Palma, and ER the field catalogue of Dr. Enrique Rodríguez-Serrano. (DOC) [file pone.0180231.s001.doc]

**Supporting Information (S1)**

**Mountaintops phylogeography: a case study using small mammals from the Andes and the Coast of central Chile.**

R. Eduardo Palma, Pablo Gutiérrez-Tapia, Juan F. González and Dusan Boric-Bargetto

Specimens analyzed, sampling locations, geographic coordinates, GenBank accesses for both genes, and altitude for each of the locations analyzed. All localities analyzed are in central Chile, please see the study area map (Fig. 1). The NK number is a special field catalog number for tissues used by the Colección de Flora y Fauna Patricio Sanchez Reyes, Departamento de Ecología, Pontificia Universidad Católica de Chile, Santiago, Chile, and by the Museum of Southwestern Biology, University of New Mexico, USA; UCK is the new tissue number collection used by the Colección de Flora y Fauna Patricio Sanchez Reyes, Departamento de Ecología, Pontificia Universidad Católica de Chile; EP is the field catalogue of Dr. R. Eduardo Palma, and ER the field catalogue of Dr. Enrique Rodríguez-Serrano.

| Species | Catalogue Number | Locality | Initials | Latitude (S) | Longitude (W) | GenBank (DLOOP) | GenBank (FGB) | altitude (m) |
| --- | --- | --- | --- | --- | --- | --- | --- | --- |
|  |  |  |  |  |  |  |  |  |
| *A. olivacea* | NK95341 | SC Apoquindo | NK95341SCApoq | 33˚ 24' 13" | 70˚ 29' 01" | AY840040 | KT600240 | 1130 |
| *A. olivacea* | NK95549 | SC Apoquindo | NK95549SCApoq | 33˚ 24' 13" | 70˚ 29' 01" | AY840041 | KT600241 | 1130 |
| *A. olivacea* | NK95671 | SC Apoquindo | NK95671SCApoq | 33˚ 24' 13" | 70˚ 29' 01" | AY840042 | KT600242 | 1130 |
| *A. olivacea* | NK95672 | SC Apoquindo | NK95672SCApoq | 33˚ 24' 13" | 70˚ 29' 01" | AY840043 |  | 1130 |
| *A. olivacea* | NK95720 | SC Apoquindo | NK95720SCApoq | 33˚ 24' 13" | 70˚ 29' 01" | AY840044 |  | 1130 |
| *A. olivacea* | NK95812 | SC Apoquindo | NK95812SCApoq | 33˚ 24' 13" | 70˚ 29' 01" | AY840045 | KT600243 | 1130 |
| *A. olivacea* | NK96309 | SC Apoquindo | NK96309SCApoq | 33˚ 24' 13" | 70˚ 29' 01" | AY840046 |  | 1130 |
| *A. olivacea* | NK96348 | SC Apoquindo | NK96348SCApoq | 33˚ 24' 13" | 70˚ 29' 01" | AY840047 | KT600244 | 1130 |
| *A. olivacea* | NK104631 | SC Apoquindo | NK104631SCApoq | 33˚ 24' 13" | 70˚ 29' 01" | AY840048 | KT600245 | 1130 |
| *A. olivacea* | NK105427 | SC Apoquindo | NK105427SCApoq | 33˚ 24' 13" | 70˚ 29' 01" | AY840049 |  | 1130 |
| *A. olivacea* | NK106136 | El Roble | NK106136ElRoble | 32˚ 58' 34" | 71˚ 00' 50" | AY840036 |  | 2198 |
| *A. olivacea* | NK106138 | El Roble | NK106138ElRoble | 32˚ 58' 34" | 71˚ 00' 50" | KT600174 |  | 2198 |
| *A. olivacea* | NK106142 | El Roble | NK106142ElRoble | 32˚ 58' 34" | 71˚ 00' 50" | AY840038 |  | 2198 |
| *A. olivacea* | NK106143 | El Roble | NK106143ElRoble | 32˚ 58' 34" | 71˚ 00' 50" | AY840039 |  | 2198 |
| *A. olivacea* | NK106144 | El Roble | NK106144ElRoble | 32˚ 58' 34" | 71˚ 00' 50" | KT600175 |  | 2198 |
| *A. olivacea* | NK106145 | El Roble | NK106145ElRoble | 32˚ 58' 34" | 71˚ 00' 50" | KT600176 |  | 2198 |
| *A. olivacea* | NK106147 | El Roble | NK106147ElRoble | 32˚ 58' 34" | 71˚ 00' 50" | KT600177 |  | 2198 |
| *A. olivacea* | NK106148 | El Roble | NK106148ElRoble | 32˚ 58' 34" | 71˚ 00' 50" | KT600178 |  | 2198 |
| *A. olivacea* | NK106149 | El Roble | NK106149ElRoble | 32˚ 58' 34" | 71˚ 00' 50" | KT600179 |  | 2198 |
| *A. olivacea* | EP492 | El Roble | EP492ElRoble | 32˚ 58' 34" | 71˚ 00' 50" | KT600180 | KT600246 | 2198 |
| *A. olivacea* | EP554 | El Roble | EP554ElRoble | 32˚ 58' 34" | 71˚ 00' 50" | KT600181 | KT600247 | 2198 |
| *A. olivacea* | EP557 | El Roble | EP557ElRoble | 32˚ 58' 34" | 71˚ 00' 50" | KT600182 | KT600248 | 2198 |
| *A. olivacea* | EP562 | El Roble | EP562ElRoble | 32˚ 58' 34" | 71˚ 00' 50" | KT600183 | KT600249 | 2198 |
| *A. olivacea* | NK96786 | R Maipú | NK96786RMaipu | 33° 29' 40.92" | 70° 53' 34.50" | KT600184 |  | 552 |
| *A. olivacea* | NK96787 | R Maipú | NK96787RMaipu | 33° 29' 40.92" | 70° 53' 34.50" | KT600185 |  | 552 |
| *A. olivacea* | NK96788 | R Maipú | NK96788RMaipu | 33° 29' 40.92" | 70° 53' 34.50" | KT600186 |  | 552 |
| *A. olivacea* | EP580 | Farellones | EP580Farellones | 33˚ 21' 36" | 70˚ 17' 28" | KT600187 | KT600250 | 2377 |
| *A. olivacea* | EP581 | Farellones | EP581Farellones | 33˚ 21' 36" | 70˚ 17' 28" | KT600188 | KT600251 | 2377 |
| *A. olivacea* | EP582 | Farellones | EP582Farellones | 33˚ 21' 36" | 70˚ 17' 28" | KT600189 | KT600252 | 2377 |
| *A. olivacea* | EP583 | Farellones | EP583Farellones | 33˚ 21' 36" | 70˚ 17' 28" | KT600190 | KT600253 | 2377 |
| *A. olivacea* | EP585 | Farellones | EP585Farellones | 33˚ 21' 36" | 70˚ 17' 28" | KT600191 |  | 2377 |
| *A. olivacea* | EP586 | Farellones | EP586Farellones | 33˚ 21' 36" | 70˚ 17' 28" | KT600192 |  | 2377 |
| *A. olivacea* | EP617 | Farellones | EP617Farellones | 33˚ 21' 36" | 70˚ 17' 28" | KT600193 |  | 2377 |
| *A. olivacea* | EP619 | Farellones | EP619Farellones | 33˚ 21' 36" | 70˚ 17' 28" | KT600194 |  | 2377 |
| *A. olivacea* | EP622 | Farellones | EP622Farellones | 33˚ 21' 36" | 70˚ 17' 28" | KT600195 |  | 2377 |
| *A. olivacea* | EP623 | Farellones | EP623Farellones | 33˚ 21' 36" | 70˚ 17' 28" | KT600196 | KT600254 | 2377 |
| *A. olivacea* | EP624 | Farellones | EP624Farellones | 33˚ 21' 36" | 70˚ 17' 28" | KT600197 | KT600255 | 2377 |
| *A. olivacea* | EP628 | Farellones | EP628Farellones | 33˚ 21' 36" | 70˚ 17' 28" | KT600198 | KT600256 | 2377 |
| *A. olivacea* | EP629 | Farellones | EP629Farellones | 33˚ 21' 36" | 70˚ 17' 28" | KT600199 |  | 2377 |
| *A. olivacea* | NK105804 | Rabuco | NK105804Rabuco | 32° 52' 11.30" | 71° 7' 20.70" | KT600200 |  | 349 |
| *A. olivacea* | NK105808 | Rabuco | NK105808Rabuco | 32° 52' 11.30" | 71° 7' 20.70" | KT600201 |  | 349 |
| *A. olivacea* | NK106153 | Melipilla | NK106153Melipilla | 33° 44' 5.49" | 71° 13' 2.38" | KT600202 |  | 147 |
| *A. olivacea* | NK106154 | Melipilla | NK106154Melipilla | 33° 44' 5.49" | 71° 13' 2.38" | KT600203 |  | 147 |
| *A. olivacea* | NK106155 | Melipilla | NK106155Melipilla | 33° 44' 5.49" | 71° 13' 2.38" | KT600204 | KT600257 | 147 |
| *A. olivacea* | NK106156 | Melipilla | NK106156Melipilla | 33° 44' 5.49" | 71° 13' 2.38" | KT600205 |  | 147 |
| *A. olivacea* | NK106157 | Melipilla | NK106157Melipilla | 33° 44' 5.49" | 71° 13' 2.38" | KT600206 | KT600258 | 147 |
| *A. olivacea* | NK106158 | Melipilla | NK106158Melipilla | 33° 44' 5.49" | 71° 13' 2.38" | KT600207 |  | 147 |
| *A. olivacea* | NK106159 | Melipilla | NK106159Melipilla | 33° 44' 5.49" | 71° 13' 2.38" | KT600208 |  | 147 |
| *A. olivacea* | NK106162 | Melipilla | NK106162Melipilla | 33° 44' 5.49" | 71° 13' 2.38" | KT600209 |  | 147 |
| *A. olivacea* | NK106163 | Melipilla | NK106163Melipilla | 33° 44' 5.49" | 71° 13' 2.38" | KT600210 |  | 147 |
| *A. olivacea* | NK106164 | Melipilla | NK106164Melipilla | 33° 44' 5.49" | 71° 13' 2.38" | KT600211 |  | 147 |
| *A. olivacea* | NK105908 | Villa Alemana | NK105908VAlemana | 33° 4' 22.29" | 71° 21' 16.70" | KT600212 |  | 254 |
| *A. olivacea* | NK105909 | Villa Alemana | NK105909VAlemana | 33° 4' 22.29" | 71° 21' 16.70" | KT600213 |  | 254 |
| *A. olivacea* | NK105910 | Villa Alemana | NK105910VAlemana | 33° 4' 22.29" | 71° 21' 16.70" | KT600214 |  | 254 |
| *A. olivacea* | NK105915 | Villa Alemana | NK105915VAlemana | 33° 4' 22.29" | 71° 21' 16.70" | KT600215 |  | 254 |
| *A. olivacea* | NK105917 | Villa Alemana | NK105917VAlemana | 33° 4' 22.29" | 71° 21' 16.70" | KT600216 |  | 254 |
| *A. olivacea* | NK105919 | Villa Alemana | NK105919VAlemana | 33° 4' 22.29" | 71° 21' 16.70" | KT600217 |  | 254 |
| *A. olivacea* | NK105920 | Villa Alemana | NK105920VAlemana | 33° 4' 22.29" | 71° 21' 16.70" | KT600218 |  | 254 |
| *A. olivacea* | NK105921 | Villa Alemana | NK105921VAlemana | 33° 4' 22.29" | 71° 21' 16.70" | KT600219 | KT600259 | 254 |
| *A. olivacea* | NK105922 | Villa Alemana | NK105922VAlemana | 33° 4' 22.29" | 71° 21' 16.70" | KT600220 | KT600260 | 254 |
| *A. olivacea* | NK105923 | Villa Alemana | NK105923VAlemana | 33° 4' 22.29" | 71° 21' 16.70" | KT600221 | KT600261 | 254 |
| *A. olivacea* | NK105924 | Villa Alemana | NK105924VAlemana | 33° 4' 22.29" | 71° 21' 16.70" | KT600222 |  | 254 |
| *A. olivacea* | NK105925 | Villa Alemana | NK105925VAlemana | 33° 4' 22.29" | 71° 21' 16.70" | KT600223 | KT600262 | 254 |
| *A. olivacea* | NK108712 | Campos Ahumada | NK108712CAhumada | 32° 40' 27.11" | 70° 31' 58.07" | KT600224 | KT600263 | 1715 |
| *A. olivacea* | NK108717 | Campos Ahumada | NK108717CAhumada | 32° 40' 27.11" | 70° 31' 58.07" | KT600225 | KT600264 | 1715 |
| *A. olivacea* | NK108719 | Campos Ahumada | NK108719CAhumada | 32° 40' 27.11" | 70° 31' 58.07" | KT600226 | KT600265 | 1715 |
| *A. olivacea* | NK108720 | Campos Ahumada | NK108720CAhumada | 32° 40' 27.11" | 70° 31' 58.07" | KT600227 | KT600266 | 1715 |
| *A. olivacea* | NK129190 | La Florida | NK129190LaFlorida | 33° 33' 48.96" | 70° 31' 54.48" | KT600228 | KT600267 | 827 |
| *A. olivacea* | NK129191 | La Florida | NK129191LaFlorida | 33° 33' 48.96" | 70° 31' 54.48" | KT600229 |  | 827 |
| *A. olivacea* | NK129192 | La Florida | NK129192LaFlorida | 33° 33' 48.96" | 70° 31' 54.48" | KT600230 |  | 827 |
| *A. olivacea* | NK129194 | La Florida | NK129194LaFlorida | 33° 33' 48.96" | 70° 31' 54.48" | KT600231 | KT600268 | 827 |
| *A. olivacea* | NK129195 | La Florida | NK129195LaFlorida | 33° 33' 48.96" | 70° 31' 54.48" | KT600232 |  | 827 |
| *A. olivacea* | NK129196 | La Florida | NK129196LaFlorida | 33° 33' 48.96" | 70° 31' 54.48" | KT600233 |  | 827 |
| *A. olivacea* | NK129198 | La Florida | NK129198LaFlorida | 33° 33' 48.96" | 70° 31' 54.48" | KT600234 |  | 827 |
| *A. olivacea* | NK129199 | La Florida | NK129199LaFlorida | 33° 33' 48.96" | 70° 31' 54.48" | KT600235 |  | 827 |
| *A. olivacea* | NK129200 | La Florida | NK129200LaFlorida | 33° 33' 48.96" | 70° 31' 54.48" | KT600236 |  | 827 |
| *A. olivacea* | NK120005 | Paine | NK120005Paine | 33° 52' 5.29" | 70° 50' 12.09" | KT600237 | KT600269 | 372 |
| *A. olivacea* | NK120007 | Paine | NK120007Paine | 33° 52' 5.29" | 70° 50' 12.09" | KT600238 |  | 372 |
| *A. olivacea* | NK120008 | Paine | NK120008Paine | 33° 52' 5.29" | 70° 50' 12.09" | KT600239 |  | 372 |
| *A. olivacea* | EP410 | Qda. de Tarapacá | EP410QTarapaca | **Outgroup** |  | AY840018 | KT600270 |  |
| *A. olivacea* | EP413 | Qda. de Tarapacá | EP413QTarapaca | **Outgroup** |  | AY840019 |  |  |
| *A. olivacea* | EP435 | Qda. de Camarones | EP435QCamarones | **Outgroup** |  | AY840016 | KT600271 |  |
| *A. olivacea* | EP438 | Qda. de Camarones | EP438QCamarones | **Outgroup** |  | AY840017 |  |  |
| *P. darwini* | NK95305 | SC Apoquindo | NK95305SCApoq | 33˚ 24' 13" | 70˚ 29' 01" | KT383308 |  | 1130 |
| *P. darwini* | NK95336 | SC Apoquindo | NK95336SCApoq | 33˚ 24' 13" | 70˚ 29' 01" | KT383309 | KT383365 | 1130 |
| *P. darwini* | NK96318 | SC Apoquindo | NK96318SCApoq | 33˚ 24' 13" | 70˚ 29' 01" | KT383310 |  | 1130 |
| *P. darwini* | NK96359 | SC Apoquindo | NK96359SCApoq | 33˚ 24' 13" | 70˚ 29' 01" | JN226699 |  | 1130 |
| *P. darwini* | NK95531 | SC Apoquindo | NK95531SCApoq | 33˚ 24' 13" | 70˚ 29' 01" | KT383311 |  | 1130 |
| *P. darwini* | NK95544 | SC Apoquindo | NK95544SCApoq | 33˚ 24' 13" | 70˚ 29' 01" | KT383312 | KT383366 | 1130 |
| *P. darwini* | NK120403 | SC Apoquindo | NK120403SCApoq | 33˚ 24' 13" | 70˚ 29' 01" | KT383313 |  | 1130 |
| *P. darwini* | NK160855 | SC Apoquindo | NK160855SCApoq | 33˚ 24' 13" | 70˚ 29' 01" | KT383314 | KT383367 | 1130 |
| *P. darwini* | EP493 | El Roble | EP493ElRoble | 32˚ 58' 34" | 71˚ 00' 50" | KT383315 |  | 2198 |
| *P. darwini* | EP544 | El Roble | EP544ElRoble | 32˚ 58' 34" | 71˚ 00' 50" | KT383316 |  | 2198 |
| *P. darwini* | EP546 | El Roble | EP546ElRoble | 32˚ 58' 34" | 71˚ 00' 50" | KT383317 |  | 2198 |
| *P. darwini* | EP548 | El Roble | EP548ElRoble | 32˚ 58' 34" | 71˚ 00' 50" | KT383318 |  | 2198 |
| *P. darwini* | EP553 | El Roble | EP553ElRoble | 32˚ 58' 34" | 71˚ 00' 50" | KT383319 | KT383368 | 2198 |
| *P. darwini* | EP558 | El Roble | EP558ElRoble | 32˚ 58' 34" | 71˚ 00' 50" | KT383320 |  | 2198 |
| *P. darwini* | EP560 | El Roble | EP560ElRoble | 32˚ 58' 34" | 71˚ 00' 50" | KT383321 |  | 2198 |
| *P. darwini* | EP566 | El Roble | EP566ElRoble | 32˚ 58' 34" | 71˚ 00' 50" | KT383322 | KT383369 | 2198 |
| *P. darwini* | EP567 | El Roble | EP567ElRoble | 32˚ 58' 34" | 71˚ 00' 50" | KT383323 | KT383370 | 2198 |
| *P. darwini* | EP569 | El Roble | EP569ElRoble | 32˚ 58' 34" | 71˚ 00' 50" | KT383324 |  | 2198 |
| *P. darwini* | EP571 | El Roble | EP571ElRoble | 32˚ 58' 34" | 71˚ 00' 50" | KT383325 | KT383371 | 2198 |
| *P. darwini* | EP575 | El Roble | EP575ElRoble | 32˚ 58' 34" | 71˚ 00' 50" | KT383326 | KT383372 | 2198 |
| *P. darwini* | NK106069 | El Roble | NK106069ElRoble | 32˚ 58' 34" | 71˚ 00' 50" | JN226723 | KT383373 | 2198 |
| *P. darwini* | NK106137 | El Roble | NK106137ElRoble | 32˚ 58' 34" | 71˚ 00' 50" | JN226724 | KT383374 | 2198 |
| *P. darwini* | NK106141 | El Roble | NK106141ElRoble | 32˚ 58' 34" | 71˚ 00' 50" | JN226725 |  | 2198 |
| *P. darwini* | NK106146 | El Roble | NK106146ElRoble | 32˚ 58' 34" | 71˚ 00' 50" | JN226726 |  | 2198 |
| *P. darwini* | EP563 | Farellones | EP563Farellones | 33˚ 21' 36" | 70˚ 17' 28" | KT383327 |  | 2377 |
| *P. darwini* | EP587 | Farellones | EP587Farellones | 33˚ 21' 36" | 70˚ 17' 28" | KT383328 |  | 2377 |
| *P. darwini* | EP588 | Farellones | EP588Farellones | 33˚ 21' 36" | 70˚ 17' 28" | KT383329 |  | 2377 |
| *P. darwini* | EP606 | Farellones | EP606Farellones | 33˚ 21' 36" | 70˚ 17' 28" | KT383330 | KT383375 | 2377 |
| *P. darwini* | EP614 | Farellones | EP614Farellones | 33˚ 21' 36" | 70˚ 17' 28" | KT383331 | KT383376 | 2377 |
| *P. darwini* | EP621 | Farellones | EP621Farellones | 33˚ 21' 36" | 70˚ 17' 28" | KT383332 |  | 2377 |
| *P. darwini* | EP625 | Farellones | EP625Farellones | 33˚ 21' 36" | 70˚ 17' 28" | KT383333 | KT383377 | 2377 |
| *P. darwini* | EP626 | Farellones | EP626Farellones | 33˚ 21' 36" | 70˚ 17' 28" | KT383334 | KT383378 | 2377 |
| *P. darwini* | EP630 | Farellones | EP630Farellones | 33˚ 21' 36" | 70˚ 17' 28" | KT383335 |  | 2377 |
| *P. darwini* | EP633 | Farellones | EP633Farellones | 33˚ 21' 36" | 70˚ 17' 28" | KT383336 |  | 2377 |
| *P. darwini* | NK108713 | Campos Ahumada | NK108713CAhumada | 32° 40' 27.11" | 70° 31' 58.07" | KT383337 |  | 1715 |
| *P. darwini* | NK108714 | Campos Ahumada | NK108714CAhumada | 32° 40' 27.11" | 70° 31' 58.07" | KT383338 | KT383379 | 1715 |
| *P. darwini* | NK108715 | Campos Ahumada | NK108715CAhumada | 32° 40' 27.11" | 70° 31' 58.07" | KT383339 |  | 1715 |
| *P. darwini* | NK108716 | Campos Ahumada | NK108716CAhumada | 32° 40' 27.11" | 70° 31' 58.07" | KT383340 |  | 1715 |
| *P. darwini* | NK108718 | Campos Ahumada | NK108718CAhumada | 32° 40' 27.11" | 70° 31' 58.07" | KT383341 | KT383380 | 1715 |
| *P. darwini* | NK108721 | Campos Ahumada | NK108721CAhumada | 32° 40' 27.11" | 70° 31' 58.07" | KT383342 | KT383381 | 1715 |
| *P. darwini* | NK108722 | Campos Ahumada | NK108722CAhumada | 32° 40' 27.11" | 70° 31' 58.07" | KT383343 | KT383382 | 1715 |
| *P. darwini* | NK108723 | Campos Ahumada | NK108723CAhumada | 32° 40' 27.11" | 70° 31' 58.07" | KT383344 |  | 1715 |
| *P. darwini* | EP539 | La Campana | EP539LaCampana | 32˚ 57' 42" | 71˚ 07' 37" | KT383345 | KT383383 | 1258 |
| *P. darwini* | EP540 | La Campana | EP540LaCampana | 32˚ 57' 42" | 71˚ 07' 37" | KT383346 | KT383384 | 1258 |
| *P. darwini* | EP542 | La Campana | EP542LaCampana | 32˚ 57' 42" | 71˚ 07' 37" | KT383347 | KT383385 | 1258 |
| *P. darwini* | UCK156 | Cantillana | UCK156Cantillana | 33˚ 55' 40.95" | 70˚ 57' 49.9" | KT383348 |  | 2000 |
| *P. darwini* | UCK157 | Cantillana | UCK157Cantillana | 33˚ 55' 40.95" | 70˚ 57' 49.9" | KT383349 |  | 2000 |
| *P. darwini* | UCK158 | Cantillana | UCK158Cantillana | 33˚ 55' 40.95" | 70˚ 57' 49.9" | KT383350 |  | 2000 |
| *P. darwini* | UCK159 | Cantillana | UCK159Cantillana | 33˚ 55' 40.95" | 70˚ 57' 49.9" | KT383351 |  | 2000 |
| *P. darwini* | UCK160 | Cantillana | UCK160Cantillana | 33˚ 55' 40.95" | 70˚ 57' 49.9" | KT383352 |  | 2000 |
| *P. darwini* | UCK162 | Cantillana | UCK162Cantillana | 33˚ 55' 40.95" | 70˚ 57' 49.9" | KT383353 | KT383386 | 2000 |
| *P. darwini* | UCK163 | Cantillana | UCK163Cantillana | 33˚ 55' 40.95" | 70˚ 57' 49.9" | KT383354 | KT383387 | 2000 |
| *P. darwini* | UCK164 | Cantillana | UCK164Cantillana | 33˚ 55' 40.95" | 70˚ 57' 49.9" | KT383355 |  | 2000 |
| *P. darwini* | UCK165 | Cantillana | UCK165Cantillana | 33˚ 55' 40.95" | 70˚ 57' 49.9" | KT383356 |  | 2000 |
| *P. darwini* | UCK166 | Cantillana | UCK166Cantillana | 33˚ 55' 40.95" | 70˚ 57' 49.9" | KT383357 |  | 2000 |
| *P. darwini* | UCK167 | Cantillana | UCK167Cantillana | 33˚ 55' 40.95" | 70˚ 57' 49.9" | KT383358 | KT383388 | 2000 |
| *P. darwini* | UCK168 | Cantillana | UCK168Cantillana | 33˚ 55' 40.95" | 70˚ 57' 49.9" | KT383359 |  | 2000 |
| *P. darwini* | UCK177 | Chicauma | UCK177Chicauma | 33˚ 16' 59" | 70˚ 58' 14" | KT383360 |  | 1905 |
| *P. darwini* | UCK178 | Chicauma | UCK178Chicauma | 33˚ 16' 59" | 70˚ 58' 14" | KT383361 | KT383389 | 1905 |
| *P. darwini* | UCK181 | Chicauma | UCK181Chicauma | 33˚ 16' 59" | 70˚ 58' 14" | KT383362 | KT383390 | 1905 |
| *P. darwini* | UCK183 | Chicauma | UCK183Chicauma | 33˚ 16' 59" | 70˚ 58' 14" | KT383363 | KT383391 | 1905 |
| *P. darwini* | NK108791 | El Canelo | NK108791Elcanelo | 33° 33' 21.89" | 70° 27' 15.99" | KT383364 |  | 1200 |
| *P. magister* | ER4 | Rio Loa | 4Pmagister | **Outgroup** |  | JN226733 | KT383392 |  |
| *P. magister* | ER5 | Rio Loa | 5Pmagister | **Outgroup** |  | JN226734 | KT383393 |  |
