# Supplementary material for: Mountaintops phylogeography: A case study using small mammals from the Andes and the coast of central Chile
Source: PLoS One. 2017 Jul 3;12(7):e0180231. doi: 10.1371/journal.pone.0180231 (PMC5495339; doi:10.1371/journal.pone.0180231)
Supplement: S2 Table — Haplotypes recovered for Abrothrix olivacea by sequencing the D-LOOP and the FGB genes (for the latter, we show the haplotypes of the concatenated D-LOOP and FGB matrix). We show the haplotype number, the frequency of that haplotype and the voucher with the abbreviation of the geographic locality (see S1 Table for complete details of each locality). The NK number is a special field catalog number for tissues used by the Colección de Flora y Fauna Patricio Sanchez Reyes, Departamento de Ecología, Pontificia Universidad Católica de Chile, Santiago, Chile, and by the Museum of Southwestern Biology, University of New Mexico, USA; UCK is the new tissue number collection used by the Colección de Flora y Fauna Patricio Sanchez Reyes, Departamento de Ecología, Pontificia Universidad Católica de Chile; EP is the field catalogue of Dr. R. Eduardo Palma, and ER the field catalogue of Dr. Enrique Rodríguez-Serrano. (DOCX) [file pone.0180231.s002.docx]

**Supporting Information (S2)**

**Mountaintops phylogeography: a case study using small mammals from the Andes and the Coast of central Chile.**

R. Eduardo Palma, Pablo Gutiérrez-Tapia, Juan F. González and Dusan Boric-Bargetto

Haplotypes recovered for *Abrothrix olivacea* by sequencing the D-LOOP and the FGB genes (for the latter, we show the haplotypes of the concatenated D-LOOP and FGB matrix). We show the haplotype number, the frequency of that haplotype and the voucher with the abbreviation of the geographic locality (see Appendix S1 for complete details of each locality). The NK number is a special field catalog number for tissues used by the Colección de Flora y Fauna Patricio Sanchez Reyes, Departamento de Ecología, Pontificia Universidad Católica de Chile, Santiago, Chile, and by the Museum of Southwestern Biology, University of New Mexico, USA; UCK is the new tissue number collection used by the Colección de Flora y Fauna Patricio Sanchez Reyes, Departamento de Ecología, Pontificia Universidad Católica de Chile; EP is the field catalogue of Dr. R. Eduardo Palma, and ER the field catalogue of Dr. Enrique Rodríguez-Serrano.

| Gene | Haplotype number | Frequency | Voucher & localities |
| --- | --- | --- | --- |
|  |  |  |  |
| D-LOOP | 1 | 33 | NK95341SCApoq NK95720SCApoq NK95812SCApoq NK106136ElRoble NK106138ElRoble NK106142ElRoble NK106147ElRoble |
|  |  |  | NK106148ElRoble NK106149ElRoble EP557ElRoble NK96786RMaipu NK96788RMaipu NK105808Rabuco NK106153Melipilla |
|  |  |  | NK106154Melipilla NK106156Melipilla NK106158Melipilla NK106163Melipilla NK105908VAlemana NK105915VAlemana |
|  |  |  | NK105919VAlemana NK105922VAlemana NK129190LaFlorida NK129191LaFlorida NK129192LaFlorida NK129194LaFlorida |
|  |  |  | NK129195LaFlorida NK129196LaFlorida NK129198LaFlorida NK129199LaFlorida NK129200LaFlorida NK120005Paine |
|  |  |  | NK120007Paine |
| D-LOOP | 2 | 1 | NK95549SCApoq |
| D-LOOP | 3 | 1 | NK95671SCApoq |
| D-LOOP | 4 | 8 | NK95672SCApoq NK96309SCApoq NK96348SCApoq NK104631SCApoq EP580Farellones EP582Farellones EP585Farellones |
|  |  |  | EP619Farellones |
| D-LOOP | 5 | 2 | NK105427SCApoq EP623Farellones |
| D-LOOP | 6 | 2 | NK106143ElRoble NK96787RMaipu |
| D-LOOP | 7 | 2 | NK106144ElRoble NK106164Melipilla |
| D-LOOP | 8 | 1 | NK106145ElRoble |
| D-LOOP | 9 | 1 | EP492ElRoble |
| D-LOOP | 10 | 2 | EP554ElRoble EP562ElRoble |
| D-LOOP | 11 | 2 | EP581Farellones EP624Farellones |
| D-LOOP | 12 | 2 | EP583Farellones EP622Farellones |
| D-LOOP | 13 | 2 | P586Farellones EP628Farellones |
| D-LOOP | 14 | 2 | EP617Farellones EP629Farellones |
| D-LOOP | 15 | 2 | EP410QTarapaca EP413QTarapaca |
| D-LOOP | 16 | 2 | EP435QCamarones EP438QCamarones |
| D-LOOP | 17 | 1 | NK105804Rabuco |
| D-LOOP | 18 | 1 | NK106155Melipilla |
| D-LOOP | 19 | 4 | NK106157Melipilla NK106159Melipilla NK105910VAlemana NK105923VAlemana |
| D-LOOP | 20 | 1 | NK106162Melipilla |
| D-LOOP | 21 | 4 | NK105909VAlemana NK105920VAlemana NK105921VAlemana NK120008Paine |
| D-LOOP | 22 | 2 | NK105917VAlemana NK105925VAlemana |
| D-LOOP | 23 | 1 | NK105924VAlemana |
| D-LOOP | 24 | 1 | NK108712CAhumada |
| D-LOOP | 25 | 2 | NK108717CAhumada NK108720CAhumada |
| D-LOOP | 26 | 1 | NK108719CAhumada |
|  |  |  |  |
| D-LOOP-FGB | 1 | 3 | NK95341SCApoq NK105922VAlemana NK120005Paine |
| D-LOOP-FGB | 2 | 1 | NK95549SCApoq |
| D-LOOP-FGB | 3 | 1 | NK95671SCApoq |
| D-LOOP-FGB | 4 | 1 | NK95812SCApoq |
| D-LOOP-FGB | 5 | 2 | NK96348SCApoq EP582Farellones |
| D-LOOP-FGB | 6 | 1 | NK104631SCApoq |
| D-LOOP-FGB | 7 | 1 | EP492ElRoble |
| D-LOOP-FGB | 8 | 2 | EP554ElRoble EP562ElRoble |
| D-LOOP-FGB | 9 | 3 | EP557ElRoble NK129190LaFlorida NK129194LaFlorida |
| D-LOOP-FGB | 10 | 1 | EP580Farellones |
| D-LOOP-FGB | 11 | 2 | EP581Farellones EP624Farellones |
| D-LOOP-FGB | 12 | 1 | EP583Farellones |
| D-LOOP-FGB | 13 | 1 | EP623Farellones |
| D-LOOP-FGB | 14 | 1 | EP628Farellones |
| D-LOOP-FGB | 15 | 1 | EP410QTarapaca |
| D-LOOP-FGB | 16 | 1 | EP435QCamarones |
| D-LOOP-FGB | 17 | 1 | NK106155Melipilla |
| D-LOOP-FGB | 18 | 1 | NK106157Melipilla |
| D-LOOP-FGB | 19 | 1 | NK105921VAlemana |
| D-LOOP-FGB | 20 | 1 | NK105923VAlemana |
| D-LOOP-FGB | 21 | 1 | NK105925VAlemana |
| D-LOOP-FGB | 22 | 1 | NK108712CAhumada |
| D-LOOP-FGB | 23 | 2 | NK108717CAhumada NK108720CAhumada |
| D-LOOP-FGB | 24 | 1 | NK108719CAhumada |
