# Supplementary material for: Mountaintops phylogeography: A case study using small mammals from the Andes and the coast of central Chile
Source: PLoS One. 2017 Jul 3;12(7):e0180231. doi: 10.1371/journal.pone.0180231 (PMC5495339; doi:10.1371/journal.pone.0180231)
Supplement: S3 Table — Haplotypes recovered for Phyllotis darwini by sequencing the D-LOOP and the FGB genes (for the latter, we show the haplotypes of the concatenated D-LOOP and FGB matrix). We show the haplotype number, the frequency of that haplotype and the voucher with the abbreviation of the geographic locality (see S1 Table for complete details of each locality). The NK number is a special field catalog number for tissues used by the Colección de Flora y Fauna Patricio Sanchez Reyes, Departamento de Ecología, Pontificia Universidad Católica de Chile, Santiago, Chile, and by the Museum of Southwestern Biology, University of New Mexico, USA; UCK is the new tissue number collection used by the Colección de Flora y Fauna Patricio Sanchez Reyes, Departamento de Ecología, Pontificia Universidad Católica de Chile; EP is the field catalogue of Dr. R. Eduardo Palma. (DOCX) [file pone.0180231.s003.docx]

**Supporting Information (S3)**

**Mountaintops phylogeography: a case study using small mammals from the Andes and the Coast of central Chile.**

R. Eduardo Palma, Pablo Gutiérrez-Tapia, Juan F. González and Dusan Boric-Bargetto

Haplotypes recovered for *Phyllotis darwini* by sequencing the D-LOOP and the FGB genes (for the latter, we show the haplotypes of the concatenated D-LOOP and FGB matrix). We show the haplotype number, the frequency of that haplotype and the voucher with the abbreviation of the geographic locality (see Appendix S1 for complete details of each locality). The NK number is a special field catalog number for tissues used by the Colección de Flora y Fauna Patricio Sanchez Reyes, Departamento de Ecología, Pontificia Universidad Católica de Chile, Santiago, Chile, and by the Museum of Southwestern Biology, University of New Mexico, USA; UCK is the new tissue number collection used by the Colección de Flora y Fauna Patricio Sanchez Reyes, Departamento de Ecología, Pontificia Universidad Católica de Chile; EP is the field catalogue of Dr. R. Eduardo Palma.

| Gene | Haplotype # | Frequency | Voucher & localities |
| --- | --- | --- | --- |
|  |  |  |  |
| D-LOOP | 1 | 1 | NK95305SCApoq |
| D-LOOP | 2 | 1 | NK95336SCApoq |
| D-LOOP | 3 | 1 | NK96318SCApoq |
| D-LOOP | 4 | 1 | NK96359SCApoq |
| D-LOOP | 5 | 1 | NK95531SCApoq |
| D-LOOP | 6 | 1 | NK95544SCApoq |
| D-LOOP | 7 | 2 | NK120403SCApoq EP571ElRoble |
| D-LOOP | 8 | 1 | NK160855SCApoq |
| D-LOOP | 9 | 16 | EP493ElRoble EP544ElRoble EP546ElRoble EP548ElRoble EP553ElRoble EP558ElRoble EP560ElRoble |
|  |  |  | EP566ElRoble EP567ElRoble EP569ElRoble EP575ElRoble NK106137ElRoble NK106141ElRoble |
|  |  |  | NK106146ElRoble UCK177Chicauma UCK183Chicauma |
| D-LOOP | 10 | 1 | NK106069ElRoble |
| D-LOOP | 11 | 2 | EP563Farellones EP625Farellones |
| D-LOOP | 12 | 1 | EP587Farellones |
| D-LOOP | 13 | 1 | EP588Farellones |
| D-LOOP | 14 | 3 | EP606Farellones EP614Farellones EP626Farellones |
| D-LOOP | 15 | 1 | EP621Farellones |
| D-LOOP | 16 | 1 | EP630Farellones |
| D-LOOP | 17 | 1 | EP633Farellones |
| D-LOOP | 18 | 1 | NK108713CAhumada |
| D-LOOP | 19 | 2 | NK108714CAhumada NK108718CAhumada |
| D-LOOP | 20 | 2 | NK108715CAhumada NK108716CAhumada |
| D-LOOP | 21 | 1 | NK108721CAhumada |
| D-LOOP | 22 | 1 | NK108722CAhumada |
| D-LOOP | 23 | 1 | NK108723CAhumada |
| D-LOOP | 24 | 2 | EP539LaCampana EP540LaCampana |
| D-LOOP | 25 | 1 | EP542LaCampana |
| D-LOOP | 26 | 4 | UCK156Cantillana UCK157Cantillana UCK162Cantillana UCK163Cantillana |
| D-LOOP | 27 | 2 | UCK158Cantillana UCK160Cantillana |
| D-LOOP | 28 | 3 | UCK159Cantillana UCK166Cantillana UCK168Cantillana |
| D-LOOP | 29 | 1 | UCK164Cantillana |
| D-LOOP | 30 | 1 | UCK165Cantillana |
| D-LOOP | 31 | 1 | UCK167Cantillana |
| D-LOOP | 32 | 1 | UCK178Chicauma |
| D-LOOP | 33 | 1 | UCK181Chicauma |
| D-LOOP | 34 | 1 | NK108791Elcanelo |
| D-LOOP | 35 | 2 | 4Pmagister 5Pmagister |
|  |  |  |  |
|  |  |  |  |
| D-LOOP-FGB | 1 | 1 | NK95336SCApoq |
| D-LOOP-FGB | 2 | 1 | NK95544SCApoq |
| D-LOOP-FGB | 3 | 1 | NK160855SCApoq |
| D-LOOP-FGB | 4 | 5 | EP553ElRoble EP566ElRoble EP575ElRoble NK106137ElRoble UCK183Chicauma |
| D-LOOP-FGB | 5 | 1 | EP567ElRoble |
| D-LOOP-FGB | 6 | 1 | EP571ElRoble |
| D-LOOP-FGB | 7 | 1 | NK106069ElRoble |
| D-LOOP-FGB | 8 | 3 | EP606Farellones EP614Farellones EP626Farellones |
| D-LOOP-FGB | 9 | 1 | EP625Farellones |
| D-LOOP-FGB | 10 | 1 | NK108714CAhumada |
| D-LOOP-FGB | 11 | 1 | NK108718CAhumada |
| D-LOOP-FGB | 12 | 1 | NK108721CAhumada |
| D-LOOP-FGB | 13 | 1 | NK108722CAhumada |
| D-LOOP-FGB | 14 | 2 | EP539LaCampana EP540LaCampana |
| D-LOOP-FGB | 15 | 1 | EP542LaCampana |
| D-LOOP-FGB | 16 | 2 | UCK162Cantillana UCK163Cantillana |
| D-LOOP-FGB | 17 | 1 | UCK167Cantillana |
| D-LOOP-FGB | 18 | 1 | UCK178Chicauma |
| D-LOOP-FGB | 19 | 1 | UCK181Chicauma |
| D-LOOP-FGB | 20 | 2 | 4Pmagister 5Pmagister |
